# Automated Implementation of the Edinburgh Visual Gait Score (EVGS)

**DOI:** 10.3390/s25103226

**Published:** 2025-05-21

**Authors:** Ishaasamyuktha Somasundaram, Albert Tu, Ramiro Olleac, Natalie Baddour, Edward D. Lemaire

**Affiliations:** 1 Department of Mechanical Engineering, University of Ottawa, Ottawa, ON K1N 6N5, Canada; nbaddour@uottawa.ca; 2 Department of Surgery, Division of Neurosurgery, Children’s Hospital of Eastern Ontario, Ottawa, ON K1H 8L1, Canada; atu@cheo.on.ca; 3 Department of Surgery, University of Ottawa, Ottawa, ON K1N 6N5, Canada; 4 Department of Neuroorthopeadics, Division of Surgery, Nicolas Avellaneda Hospital, San Miguel de Tucuman 4000, Argentina; ortopediainfantiltucuman@gmail.com; 5 Faculty of Engineering, University of Ottawa, Ottawa, ON K1H 8M2, Canada

**Keywords:** gait analysis, cerebral palsy, Edinburgh visual gait score (EVGS), pose estimation, OpenPose, automated scoring, real-world patient data, smartphone, video analysis

## Abstract

The Edinburgh Visual Gait Score (EVGS) is a commonly used clinical scale for assessing gait abnormalities, providing insight into diagnosis and treatment planning. However, its manual implementation is resource-intensive and requires time, expertise, and a controlled environment for video recording and analysis. To address these issues, an automated approach for scoring the EVGS was developed. Unlike past methods dependent on controlled environments or simulated videos, the proposed approach integrates pose estimation with new algorithms to handle operational challenges present in the dataset, such as minor camera movement during sagittal recordings, slight zoom variations in coronal views, and partial visibility (e.g., missing head) in some videos. The system uses OpenPose for pose estimation and new algorithms for automatic gait event detection, stride segmentation, and computation of the 17 EVGS parameters across the sagittal and coronal planes. Evaluation of gait videos of patients with cerebral palsy showed high accuracy for parameters such as hip and knee flexion but a need for improvement in pelvic rotation and hindfoot alignment scoring. This automated EVGS approach can minimize the workload for clinicians through the introduction of automated, rapid gait analysis and enable mobile-based applications for clinical decision-making.

## 1. Introduction

Markerless pose estimation is a method for estimating the position and orientation of a human and/or object without using a physical marker. Such an approach can be utilized in robotics, augmented reality, fitness, sports analytics, yoga training, and other movement-related areas [1,2,3,4]. Pose estimation methods are diverse and include model-based, feature-based, and deep learning-based methods [5].

Recent advances have improved two-dimensional and three-dimensional human pose estimation methodologies. Deep learning architectures, including Convolutional Neural Networks, DeepPose, OpenPose, and optimization techniques have revolutionized this field [6,7]. In addition, research has been extended to include object pose estimation, where deep learning techniques outperform other techniques with regard to accuracy and robustness [8].

OpenPose is an open-source two-dimensional pose estimation model that identifies body and facial keypoints from images or video [9,10]. OpenPose has been evaluated for tracking infant reaching movements [9] and pose estimation for hurdling athletics [10]. In a different study, OpenPose was utilized in robotic motion control and humanoid robot training using keypoint extraction, motion feature analysis, and activity recognition [11]. The flexibility of OpenPose makes it suitable for numerous applications, such as sports analysis, robotics, and automation, with research to recognize postures when exercising, analyze vector geometry, and provide personalized feedback in terms of suggestions and correction [12]. Healthcare applications include quantitative measurements of motor skills, motion analysis, and rehabilitation follow-up [13]. As development continues, better accuracy and robustness in pose estimation techniques are expected [5].

Gait analysis is an essential tool for understanding human locomotion and diagnosing movement pathologies. In particular, gait analysis is a pillar in the management and treatment of ambulatory children with cerebral palsy (CP). Recent developments have highlighted the utility of clinical gait evaluation with visual assessment [14].

Visual Gait Analysis (VGA) is valuable for assessing gait in children with CP, providing information on functional mobility when other advanced tools are not available [15]. The Edinburgh Visual Gait Score (EVGS) is a validated and objective gait assessment tool, specifically for people with CP [16]. The EVGS has modest correlations with functional mobility tests, including the Timed Up and Go Test and Gross Motor Function Classification System [17]. Furthermore, the EVGS links impaired selective voluntary motor control in CP children to key gait parameters, including foot clearance and maximum ankle dorsiflexion during the swing phase [18]. Moderate correlations between the EVGS and trunk motions highlight the utility of EVGS to capture functional features of gait modulations [18]. The EVGS can increase the usability and accessibility of gait analysis, such that analyses can be practically implemented in a variety of clinical environments [19]. Robinson et al. [20] determined the minimal clinically important difference (MCID) of EVGS to be 2.4, which corresponds with assessment tools based on functional observation (e.g., Gross Motor Function Classification System). These results confirm the utility of VGA, particularly EVGS, for the diagnosis and quantification of gait irregularities in CP patients.

However, EVGS use can be resource-intensive since time and expertise are required for implementation. Furthermore, a controlled environment is required for video recording and analysis. In turn, research using digital tools to calculate the EVGS is paving the way for accessible and efficient gait analysis in clinical practice. Aroojis et al. [21] showed the viability of applying smartphone slow-motion video and motion analysis software (Hudl app, ©Agile Sports Technologies Inc., Lincoln, NE, USA) to effectively implement the EVGS. While this could improve EVGS scoring, this approach does not provide automated scoring. The joint angles were calculated from the software and the EVGS was scored manually. The average time to calculate the EVGS was 24.7 min (16–55 min).

Currently, the only automated EVGS scoring system in the literature is the approach by Ramesh et al. [22]. However, several studies have identified gait scoring using algorithm-based approaches. One method, based on Rancho Observational Gait Analysis, required a three-dimensional lab and did not include automated EVGS reporting, hence limiting its accessibility [23]. Viswakumar et al. [24] proposed a low-cost markerless system that leverages a smartphone and OpenPose. This framework only supported measurement of knee flexion and extension angles making it insufficient for full EVGS automation.

Smartphone slow-motion videos and tools like Hudl allow clinicians to manually assess EVGS parameters, enhancing usability but lacking automation [21]. Ramesh et al. [22] introduced an automated EVGS method using smartphone videos and OpenPose BODY25. Gait events (foot strike, toe-off, mid-midstance, mid-midswing) were detected based on kinematic features, such as relative distances of heels/toes to the midhip keypoint.

EVGS parameters were calculated using joint angles and limb orientations derived from body keypoints. For instance, the hip angle was determined between a line perpendicular to the trunk and the thigh segment, while coronal parameters (e.g., pelvic obliquity, hindfoot valgus/varus) were assessed by measuring segment angles against the image frame.

The system, tested on able-bodied participants simulating various gait patterns, showed high correlations (r > 0.8) in 8 of the 17 EVGS parameters and moderate accuracy in others. Gait event detection accuracy was within 2–5 frames, and view detection exceeded 90% accuracy. However, since testing was performed on a purpose-built dataset of able-bodied people who performed all EVGS parameter movements, it may not capture the full variability and complexity of pathological gait. The method needs validation on clinical data to assess its performance in real-world pathological gait conditions. To be used clinically, the automated EVGS scoring system requires further developments to improve performance for videos taken in clinics of people with movement disorders.

This study presents and evaluates an efficient system for automatic event detection, stride segmentation, and EVGS scoring from 2D videos of patient gait. These real-world videos present technical issues that are analogous to the problems that clinicians must address when managing patient video data in practice. An efficient and accurate system could alleviate the clinician’s workload by reducing or eliminating the need for manual intervention when scoring EVGS trials. Such an automated approach would also enhance patient accessibility to gait analysis since video could be captured at the point of patient contact.

## 2. Algorithm Development

To achieve automated EVGS analysis, the proposed system must identify the appropriate video frames for analysis, and then apply a series of rules to generate scores for each parameter. Specific gait events in walking videos must be detected, and an algorithm capable of scoring EVGS parameters must be implemented. Building upon the automated EVGS scoring system presented by Ramesh et al. [22], the proposed approach involves a sequential pipeline that includes pose estimation, plane detection, movement direction determination, gait event identification, stride segmentation, and the algorithmic computation of EVGSs.

New algorithms were developed in the paper for plane detection, direction detection (for both sagittal and coronal), error handling for incomplete or missing foot strike data, and poor keypoints since EVGS scoring issues were found when using clinical videos that did not have the same experimental rigor as the able-bodied person dataset used for initial validation. More robust methods were required to deal with camera zooming, multiple people in the frame, and other anomalies that adversely affected automated EVGS scoring. Details on these new algorithms are in the subsequent sections.

**Figure 1:**
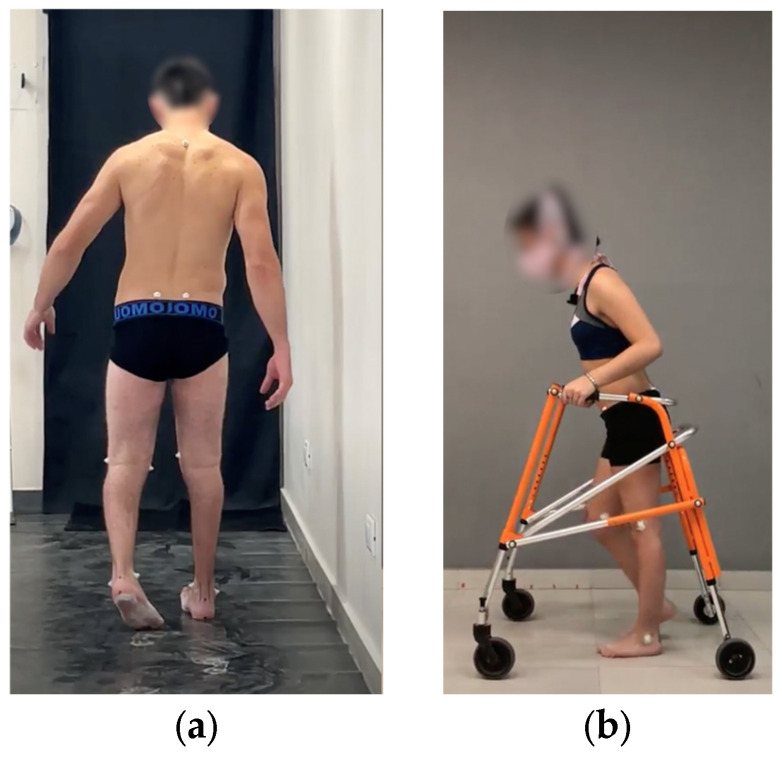
Sample videos in (**a**) coronal and (**b**) sagittal planes.

A video dataset of patients (aged 6–40, 34 males and 16 females) with cerebral palsy was obtained from Sanatorio del Norte Medical Center and CINEA Medical Center in Tucumán, Argentina, and used for EVGS algorithm development and evaluation. The set provided sagittal and coronal view gait videos for 230 patients with varying degrees of mobility dysfunction. For sagittal videos, the camera was parallel to the direction of motion. For the coronal plane, the camera was in front of the participant. The camera was positioned at hip height for the sagittal plane and the camera was placed on the ground for the coronal plane. Videos were recorded at 60 Hz and captured in a closed environment with good lighting and the camera height was consistent in all trials (Figure 1).

### 2.1. Qualitative Video Assessment

To work with appropriate videos for algorithm development, the Sanatorio del Norte and CINEA patient video sets were qualitatively assessed to select videos that were suitable for automated EVGS development, including sagittal and coronal views. Included videos provided a clear coronal or sagittal plane view, with full-body visibility. Videos were excluded from analysis if more than one person was in the frame, if an overhead operator could be seen, or if there was excessive zooming throughout the video. When possible, videos were cropped or trimmed to isolate the patient in the video (i.e., remove sections where people were helping the patient, or an overhead cameraman was visible in the frame). A total of 50 patient video sets met the inclusion criteria. Twenty (20) video sets were used to develop the algorithm and 30 video sets were used to validate the algorithm.

### 2.2. Video Processing

**Figure 2:**
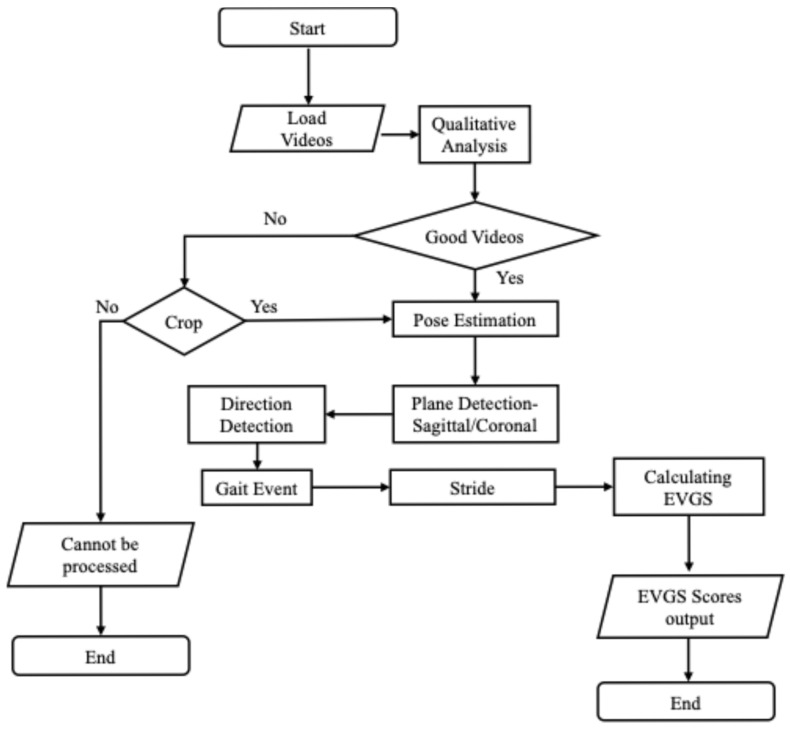
Flowchart of the overall algorithm.

**Figure 3:**
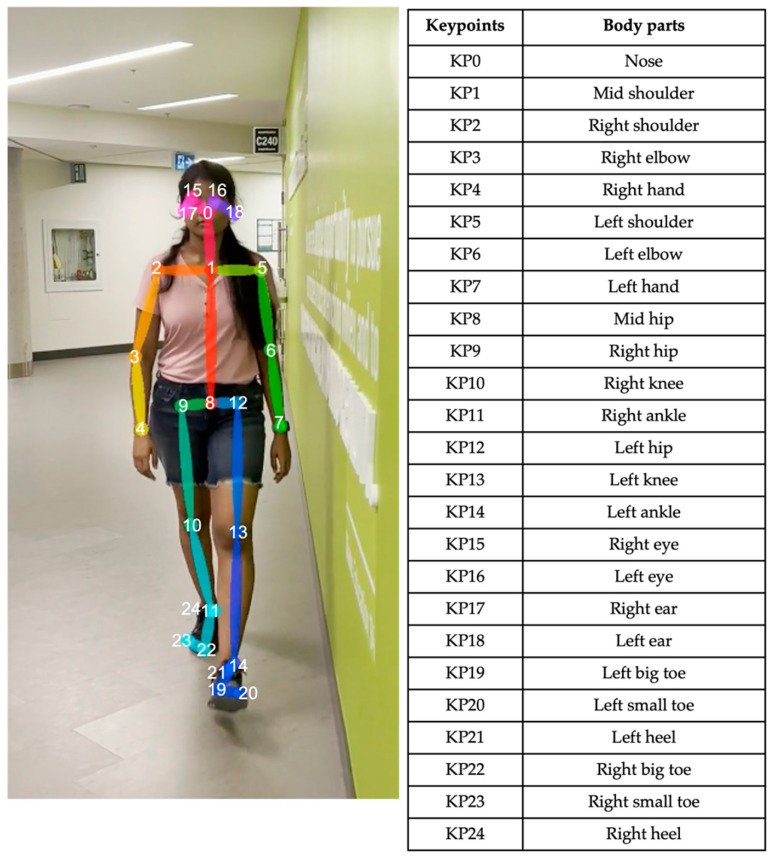
OpenPose human pose estimation using BODY25 model [22].

Figure 2 shows the general methodology for the automated algorithm. The process begins with a qualitative assessment of the patient sets, using the criteria in Section 2.1. Video sets that do not meet these standards were further reviewed to determine if they could be adjusted for usability. Video sets that could not be adequately cropped or trimmed were rejected from further analysis. The next step involves pose estimation using OpenPose, a well-established pose estimation model [25,26,27]. The OpenPose BODY25 model detects 2D body keypoints on the head, trunk, and limbs (Figure 3). Python (v3.10) was used to perform the computations.

Once pose estimation is completed, the system performs direction detection to determine if the person is walking left to right or right to left in the sagittal plane or moving towards or away from the camera in the coronal plane. Subsequently, the algorithm identifies whether the video is recorded from a sagittal (side) or coronal (front/back) perspective. Following direction and perspective identification, the system detects gait events, such as heel strikes and toe-offs. Stride detection builds on gait event detection by identifying complete strides, from one heel strike to the next heel strike on the same foot. All subsequent processing is performed on each stride. Following stride detection, EVGSs for each stride are calculated for 17 parameters (12 sagittal and 5 coronal). Then, the EVGS algorithm is applied to the pose estimation and gait event data to generate scores for each video set.

#### 2.2.1. Keypoint Processing

Since the raw keypoint coordinate trajectories often contain noise and sometimes outliers, the keypoint time series were filtered using a zero-phase, dual-pass, second-order Butterworth filter with a 12 Hz cut-off frequency. Two-dimensional keypoint processing was adapted from a previously established markerless Artificial Intelligence (AI) motion analysis methodology for hallways [28]. Keypoints with confidence scores below a 10% threshold were excluded, and any resulting gaps up to five frames (0.083 s) were interpolated using cubic spline interpolation [29]. For Ramesh et al. [22], low-confidence keypoints with a confidence score of less than 0.5 were detected and removed. Subsequently, missing keypoints were interpolated to provide keypoints for each frame; however, if data gaps were abnormally extended, then interpolation became impossible, leaving frames with missing keypoints.

Since EVGS parameters are calculated from specific sections of the video, such as at heel strike or midstance, an updated code was implemented before calculating EVGS parameters to verify that all keypoints necessary for parameter calculations are available. If the essential keypoints for a parameter calculation are incomplete, the code exits and does not move forward with EVGS scoring. This step avoids inappropriately discarding videos with missing keypoints in areas where they do not affect parameter calculations.

#### 2.2.2. Coronal/Sagittal Plane Detection

**Figure 4:**
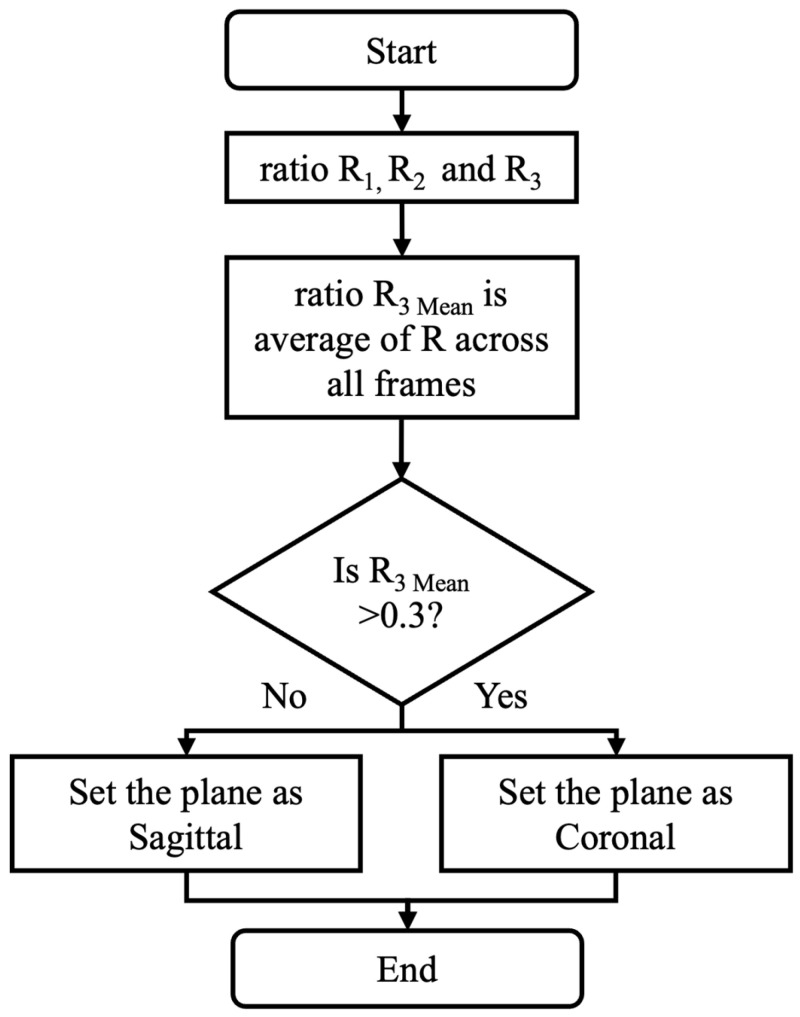
Flowchart depicting the detection of sagittal/coronal plane view.

As shown in Figure 4, torso ratios are used to distinguish the sagittal view from the coronal view videos. These ratios are based on the spatial ratios between the shoulders and hips, since variations in these ratios can provide information about the body’s orientation with respect to the camera. The torso ratios are used for identifying the plane because they are independent of camera zoom in the video data.

For each frame, R_1_ is the ratio of the distance between the left shoulder and left hip to the distance between the right and left shoulders (Equation (1)).

(1) $$R_{1}=\frac{\sqrt{{\left(x_{\mathrm{left}\_\mathrm{shoulder}}-x_{\mathrm{left}\_\mathrm{hip}}\right)}^{2}+{\left(y_{\mathrm{left}\_\mathrm{shoulder}}-y_{\mathrm{left}\_\mathrm{hip}}\right)}^{2}}}{\sqrt{{\left(x_{\mathrm{right}\_\mathrm{shoulder}}-x_{\mathrm{left}\_\mathrm{shoulder}}\right)}^{2}+{\left(y_{\mathrm{right}\_\mathrm{shoulder}}-y_{\mathrm{left}\_\mathrm{shoulder}}\right)}^{2}}}$$

For each frame, R_2_ is the ratio of the distance between the right and left shoulders to the distance between the right shoulder and right hip, assessing the upper body width relative to the vertical length of the right side (Equation (2)).

(2) $$R_{2}=\frac{\sqrt{{\left(x_{\mathrm{right}\_\mathrm{shoulder}}-x_{\mathrm{left}\_\mathrm{shoulder}}\right)}^{2}+{\left(y_{\mathrm{right}\_\mathrm{shoulder}}-y_{\mathrm{left}\_\mathrm{shoulder}}\right)}^{2}}}{\sqrt{{\left(x_{\mathrm{right}\_\mathrm{shoulder}}-x_{\mathrm{right}\_\mathrm{hip}}\right)}^{2}+{\left(y_{\mathrm{right}\_\mathrm{shoulder}}-y_{\mathrm{right}\_\mathrm{hip}}\right)}^{2}}}$$

Finally, R_3_ is calculated as the ratio of the previous two ratios:

(3) $$R_{3}=\frac{R_{1}}{R_{2}}$$

The (per-video) mean of the (per-frame) R_3_ values is calculated and used for the “coronal or sagittal” decision.

The average R_3_ value for a video (R_3 Mean_) is calculated by averaging the R_3_ values from all frames in the video. A decision rule is used for coronal/sagittal plane classification, such that if the R_3 Mean_ is greater than 0.3 the view is classified as sagittal. If the R_3 Mean_ is less than or equal to 0.3, the view is coronal. This threshold was determined based on an analysis of 20 videos used during algorithm development, where the threshold was selected to maximize classification accuracy across a range of body types and video angles.

#### 2.2.3. Direction Detection

**Figure 5:**
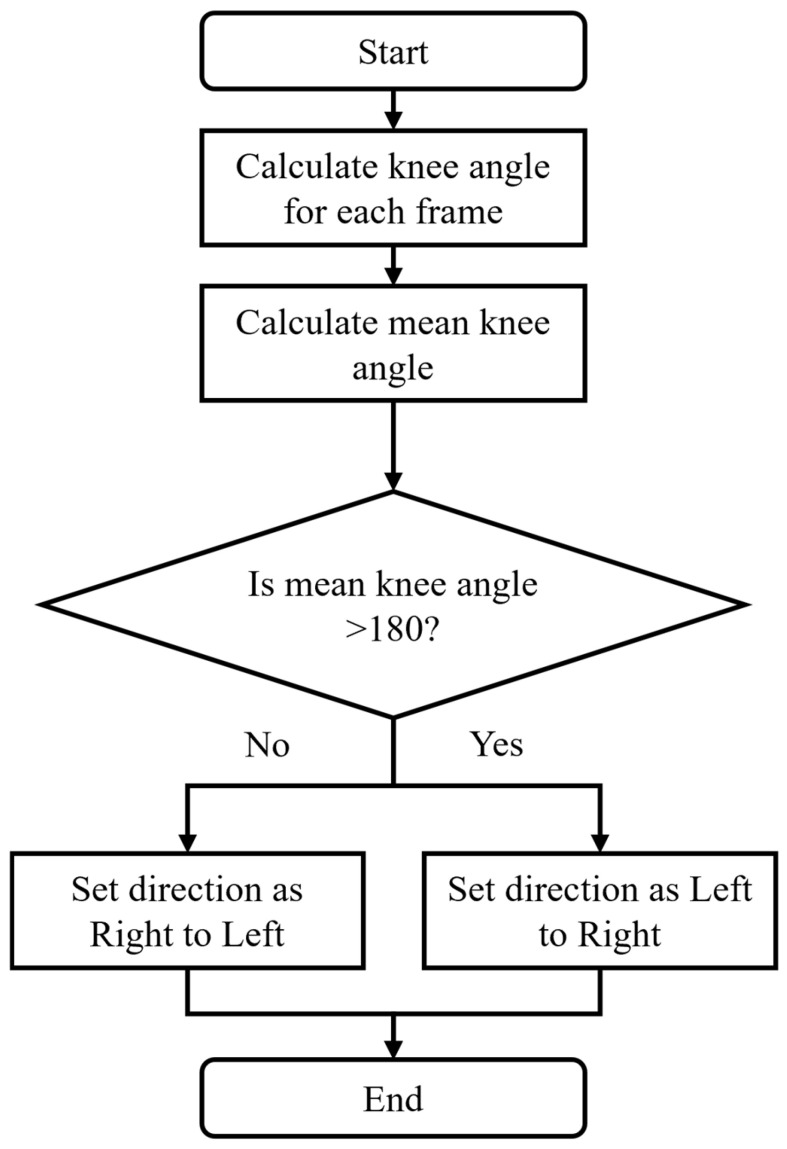
Flowchart to identify direction of movement in the sagittal plane.

In the sagittal plane, the process for detecting movement direction (Figure 5) starts by calculating the knee angle for each video frame, using the hip, knee, and ankle joint coordinates (Equation (4)). The numpy $arctan2\left(y,\;x\right)$ function computes the angle between the positive x-axis and a vector from the origin to the point (*x*,*y*) in two-dimensional Cartesian space. Unlike the standard arctangent function arctan(y/x), arctan2 considers the signs of both (x) and (y) to determine the correct quadrant of the angle, making it more robust for applications involving directional calculations. In (Equation (4)), the first arctan2 term calculates the angle of the line segment between the knee and the ankle, while the second arctan2 term calculates the angle of the line segment between the knee and the hip. Subtracting these angles gives the relative joint angle at the knee.

Let A_rh_rk_ and A_ra_rk_ represent the vectors between the hip, knee, and ankle. The directional angle $\theta_{i}$ for each frame i is calculated as

(4) $$\theta_{i}=arctan2(y_{ankle}-y_{knee},x_{ankle}-x_{knee})-arctan2(y_{hip}-y_{knee},x_{hip}-x_{knee})$$

Knee angles are calculated for the right leg, and the average angle for the right leg is calculated for the entire video.

A decision rule based on threshold values is used to classify the movement direction. A threshold of 180° was selected from empirical observation of the development video set. If the Mean Knee Angle is >180°, then the direction of movement is right to left. If the Mean Knee Angle is ≤180°, the motion is classified as left to right.

**Figure 6:**
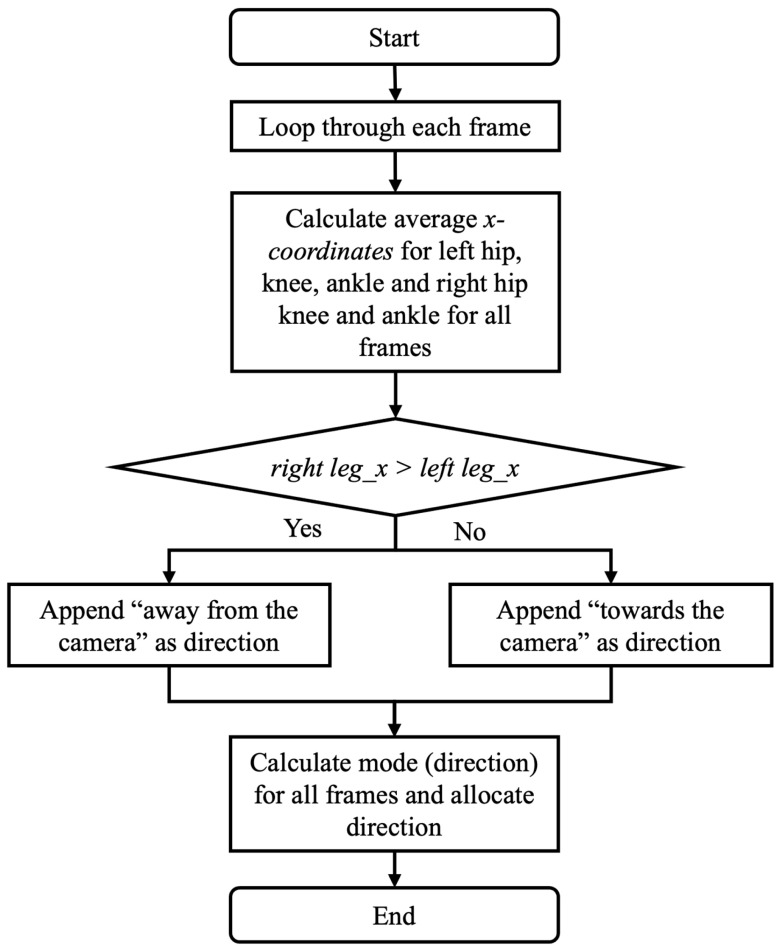
Flowchart to identify direction of movement in the coronal plane.

The coronal detection process is shown in Figure 6**.** For each frame in the video, x-coordinates of the hip, knee, and ankle are extracted for both legs. The right leg average is the average x-coordinate for the 3 right joints. The left leg average is the average x-coordinate for the 3 left joints. If the right leg average is greater than the left leg average, the person is walking away from the camera. If the right leg average is less than the left leg average, the person is walking towards the camera. After calculating the direction for each frame, the most frequent classification (mode) of the direction classifications for all frames is used to select the walking direction in the coronal plane. Gait event detection was calculated using the method reported by Ramesh et al. [22].

### 2.3. EVGS Parameters

The Edinburgh Visual Gait Score consists of 17 parameters for each of the 2 legs, totaling 34 parameters. Commonly, sagittal and coronal views of a patient are recorded with a handheld camera or one attached to a tripod. These videos are then manually scored by clinicians in accordance with the EVGS guidelines. During scoring, video editing software is often used to pause the video at relevant gait events for detailed analysis. Software tools can also be used to calculate joint angles and other parameters relevant to EVGS. Each EVGS parameter is scored on a 3-point ordinal scale: 0 indicates normal gait (within ±1.5 standard deviations of the mean); 1 indicates moderate deviations (1.5 to 4.5 standard deviations from the mean), and 2 indicates significant or severe deviations (more than 4.5 standard deviations from the mean) [30]. A low total score reflects fewer gait deviations. To facilitate a clear understanding and to ensure consistency in scoring, the 17 EVGS parameters were grouped according to foot events and gait cycle phases (Table 1).

Most EVGS parameters require joint angles. The ankle, knee, and hip angles were calculated using the method reported by Ramesh et al.

## 3. Evaluation

### Methods

A total of 30 patient sets from the algorithm development dataset were reserved for algorithm evaluation.

Algorithm performance for sagittal and coronal planes was assessed by comparing the algorithm results with the manual classification of movement plane and movement direction. Accuracy, sensitivity, specificity, precision, and F1 score were calculated to validate the algorithm’s effectiveness for plane and movement direction detection. One examiner reviewed all videos to extract the ground truth planes and directions.

EVGS scoring was evaluated by comparing algorithm outcomes to manual EVGSs that accompanied videos in the Sanatorio del Norte and CINEA dataset (i.e., experts at Sanatorio del Norte hospital and CINEA used sagittal, coronal, and overhead views to score the EVGS for each video). Accuracy was calculated for each of the 17 EVGS movements using (Equation (5)).

(5) $$Accuracy=\frac{Number\;of\;patients\;correctly\;classified}{Total\;number\;of\;patients}$$

Accuracy was calculated for right and left legs separately and then the accuracies were averaged between legs for each of the 17 parameters. Accuracy was the metric used to compare EVGSs. For this study, high accuracy was 85–100%, moderate accuracy was 70–85%, and less than 70% was considered low accuracy.

## 4. Results

### 4.1. Coronal/Sagittal View Detection

Coronal and sagittal view detections were correct for all patient sets (100% accuracy, 100% sensitivity, 100% specificity, 100% precision, and F1 score of 1).

### 4.2. Direction of Motion Detection

The direction of motion detection in the sagittal plane was correct for all right-to-left movements and 28 of 30 left-to-right movements (96.67% accuracy, 100% sensitivity, 93.75% specificity, 93.33% precision, and F1 score of 0.96). The two misclassifications were due to multiple candidates in the video or excessive zooming.

The direction of motion detection in the coronal plane was correct for all videos (100% accuracy, 100% sensitivity, 100% specificity, 100% precision, and F1 score of 1).

### 4.3. EVGS Scoring

**Figure 7:**
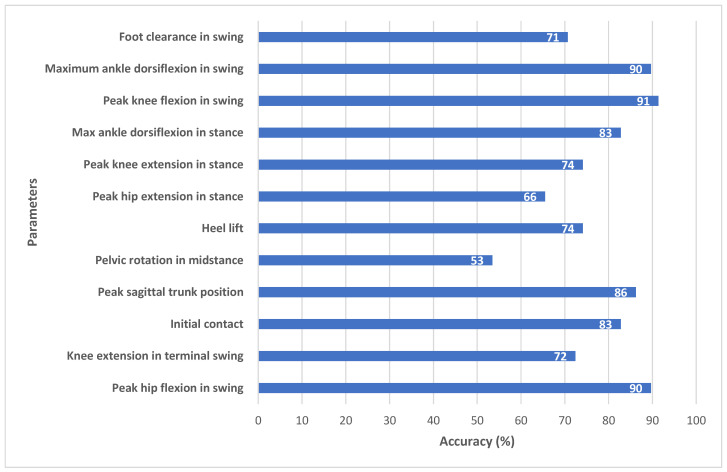
Accuracy between algorithm and ground truth for sagittal view parameters.

**Figure 8:**
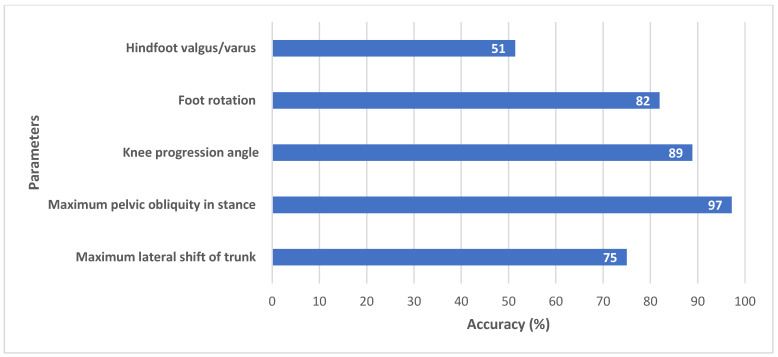
Accuracy between algorithm and ground truth for coronal view parameters.

Figure 7 (sagittal) and Figure 8 (coronal) show EVGS outcomes for accuracies averaged between the right and left legs. Peak hip flexion in swing, peak knee flexion in swing, maximum ankle dorsiflexion in swing, and maximum pelvic obliquity in stance had the best accuracies (90–97%). Initial contact, maximum ankle dorsiflexion during stance, peak sagittal trunk position, knee progression angle, and foot rotation also had high accuracies (82–89%).

Moderate accuracy (71–75%) was seen for knee extension in terminal swing, heel lift, peak knee extension in stance, peak hip extension in stance, foot clearance in swing, and maximum lateral shift in the trunk. Pelvic rotation in midstance and hindfoot valgus/varus had the lowest accuracy (52–53%).

## 5. Discussion

A viable algorithm was produced that automatically calculated the EVGSs from sagittal and coronal videos of people with gait disorders. High accuracy was achieved for most EVGS parameters, but further development could improve results for out-of-plane movements.

### 5.1. Sagittal Plane Parameters

The model demonstrated high accuracy for the majority of gait parameters, with six parameters showing high accuracy, four showing moderate accuracy, and two showing low accuracy and it demonstrated strong agreement with manual EVGS scoring. In the sagittal plane (Figure 7), the algorithm achieved high accuracy for parameters such as peak hip flexion in swing (90%), peak knee flexion in swing (91%), and maximum ankle dorsiflexion in swing (90%). Other parameters, such as initial contact (83%), maximum ankle dorsiflexion in stance (83%), and peak sagittal trunk position (86%), also fell within the high-accuracy range, suggesting that the algorithm can detect major sagittal plane movements using 2D pose estimation. Heel lift (74%) and foot clearance (71%) had moderate accuracy. Events like foot strikes and foot-offs are necessary to determine the heel lift score. Hence, this score is sensitive to small changes in these gait events. Ramesh et al. [22] reported that changes in only one frame could result in the score changing from 0 to 1. In future research, another method of scoring heel lift could be considered, where the score is less dependent on stride events and more related to inter-limb kinematics.

The foot clearance parameter had moderate accuracy. Human reviewers usually determine the foot clearance score by finding the video frame where the foot looks to be closest to the ground, and then determining the score [30]. The algorithm is slightly different, since the method does not try to calculate a ground plane, which would be used to determine the distance from the foot markers to the ground. For the algorithm, full clearance is defined as when the big toe and heel of the swing leg cross the big toe and heel of the contralateral stance leg (e.g., when walking left to right, the x-coordinates for both the left heel and big toe are greater than the x-coordinates of both the right heel and big toe) [22]. This ensures that any video (e.g., hand-held, etc.) can be processed by the automated system. These differences in approach may result in some discrepancies for cases with small foot clearances.

Knee extension in terminal swing (72%) and peak knee extension in stance (74%) had moderate accuracy (Figure 7). Out of the 30 patients, 10 had high accuracy, 10 had low accuracy, and 10 had medium accuracy. Since clinicians can find it difficult to detect small angular differences, such as between 14° and 17° (e.g., knee extension in swing angle of 5° to 15° corresponds to a score of one; angles between 16° and 30° score as two), inter-rater differences for parameters that are close to the transition between scores can occur. Since the algorithm calculates quantitative joint angles, the algorithmic approach may provide more consistent results for these cases. Further research with a larger dataset will help to confirm algorithmic scoring consistency.

Pelvic rotation in midstance (53%) and peak hip extension in stance (66%) had the lowest accuracies (Figure 7). OpenPose provides hip keypoints; however, it does not provide sufficient landmarks to determine pelvic segment orientation. Instead, the angle of pelvic rotation was calculated with a line joining the right and left hip joints relative to the y-axis (vertical) of the sagittal plane from the image coordinate system [22]. Other surrogate measures could be investigated, such as inter-hip distance changes in the coronal plane. As well, new pose detection methods that estimate depth axis could be investigated to improve automated EVGS scoring for these transverse plane movements.

Similarly, for peak hip extension, the hip angle is defined as the angle between the trunk axis and the thigh axis, since OpenPose does not provide keypoints to define a pelvis segment. The trunk axis is a line connecting the neck and midhip keypoints, while the thigh axis uses the hip and knee keypoints. Human scorers interpret hip extension as the thigh angle relative to the pelvis. Trunk angle can differ from pelvis angle during gait, especially for pathological gait where the trunk can be bent over and have a much greater range of motion. Therefore, clinician assessment can differ from the algorithm scoring.

### 5.2. Coronal Plane Parameters

Coronal view parameters had generally good accuracy of all five parameters. Maximum pelvic obliquity in stance (97%) and knee progression angle (89%) had high accuracy (Figure 8).

Foot rotation (82%) and lateral trunk shift (75%) had a moderate agreement between algorithm and ground truth. Studies have consistently shown that EVGS reliability is higher for distal segments (foot, ankle, knee) compared to proximal segments (trunk, pelvis, hip) when assessing gait abnormalities in children with cerebral palsy [21,31,32]. Therefore, some of the differences in trunk shift could be attributed to manual scoring.

**Figure 9:**
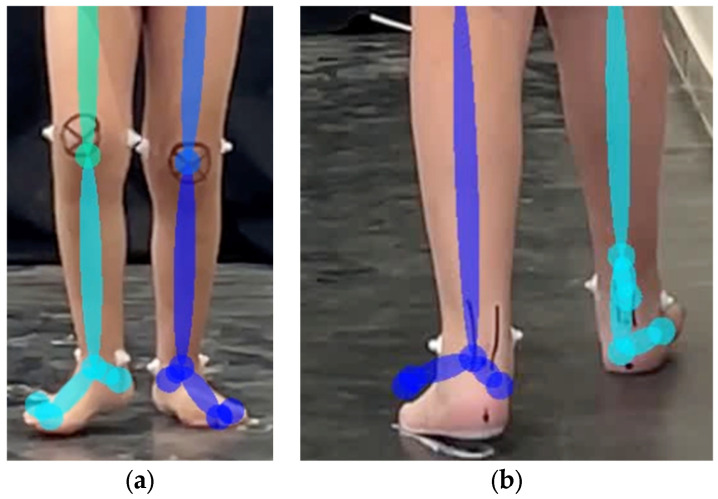
Overlapping of foot keypoints: (**a**) walking towards the camera and (**b**) walking away from the camera.

Hindfoot valgus/varus (51%) had low accuracy due to challenges in keypoint detection. In the coronal view, heel and toe keypoints tend to cluster or overlap or are occluded by the big toe keypoint (Figure 9). Ankle and heel keypoints are close to each other, and the inter-keypoint distance becomes smaller as the person walks away from the camera. Hence, these keypoints are more likely to be occluded during gait, which will result in scoring discrepancies. It is worth noting that this is not a problem with the algorithm alone, since humans also find it challenging to estimate foot keypoints under such conditions [22]. These factors can also affect foot rotation scoring.

This study has several limitations that should be acknowledged. First, participants were between 6 and 40 years of age. Encompassing both pediatric and adult populations introduces diversity that, while valuable for broader evaluation, may have affected the algorithm’s consistency. Although the EVGS was developed for children with CP, tuning the algorithm specifically for pediatric or adult participants could produce better results. Physical differences between children and adults, such as variations in body size, proportions, and movement dynamics, may not fully fit the ranges set by the EVGS for a given pediatric population. This can reduce the algorithm’s accuracy and its generalizability across different age groups. In future work, adapting algorithmic approaches for pediatric or adult populations will require larger datasets. Moreover, the algorithm was not tested against diverse patient ethnicities or scenarios involving multiple participants, which could result in errors in identifying the primary subjects or tracking their movements. The algorithm was not tested against various backgrounds or the presence of more than one person in the video, either of which could result in errors in identifying the primary person or tracking their movements.

## 6. Conclusions

This research presents an automated methodology for applying the EVGS to real patient data, testing the effectiveness of the proposed algorithm compared to expert scoring. Automatic stride detection and movement direction detection performed well in both the sagittal and coronal videos, while EVGS performance varied between parameters. Of the 17 EVGS parameters, accuracy was high for 9, moderate for 6, and low for 2 parameters. Refinements are still required for accurate classification of pelvic rotation at midstance and hindfoot valgus/varus. This automated approach is suitable for processing real-world patient video data and is promising for automating clinical gait analysis. This development increases the availability of gait evaluation and enables patient monitoring outside traditional clinical environments.

## Figures and Tables

**Table 1:** EVGS parameters [30].

	Foot Events and Gait Phases	EVGS Parameters
Sagittal	Initial contact/terminal swing	Peak hip flexion in swing (#13)
		Knee extension in terminal swing (#10)
		Initial contact (#1)
	Midstance	Peak sagittal trunk position (#16)
		Pelvic rotation in midstance (#15)
		Heel lift (#2)
	Terminal stance	Peak hip extension in stance (#12)
		Peak knee extension in stance (#9)
		Max ankle dorsiflexion in stance (#3)
	Midswing	Peak knee flexion in swing (#11)
		Maximum ankle dorsiflexion in swing (#7)
		Foot clearance in swing (#6)
Coronal	Midstance	Maximum lateral shift in trunk (#17)
		Maximum pelvic obliquity in stance (#14)
		Knee progression angle (#8)
		Foot rotation (#5)
		Hindfoot valgus/varus (#4)

## Data Availability

Data are contained within the article.

## Abbreviations

The following abbreviations are used in this manuscript:

AI	Artificial Intelligence
CHAMO	Children’s Hospital Academic Medical Organization
CP	Cerebral Palsy
EVGS	Edinburgh Visual Gait Score
MCID	Minimal Clinically Important Difference
VGA	Visual Gait Analysis
